# The effects of *FTO* gene rs9939609 polymorphism on the association between breast cancer and dietary intake

**DOI:** 10.1111/jcmm.17595

**Published:** 2022-11-20

**Authors:** Saeid Doaei, Sepideh Abdollahi, Golsa Khalatbari Mohseni, Maryam Gholamalizadeh, Mohammad Esmail Akbari, Seyed Mohammad Poorhosseini, Seyedeh Elaheh Bagheri, Soudeh Ghafouri‐Fard, Ghasem Azizi Tabesh, Alireza Moslem, Naeemeh Hasanpour Ardekanizadeh, Saeed Omidi, Azita Hekmatdoost, Mahdi Alam Rajabi, Seyed Alireza Mosavi Jarrahi, Mark O. Goodarzi

**Affiliations:** ^1^ Department of Community Nutrition, Faculty of Nutrition and Food Technology, National Nutrition and Food Technology Research Institute Shahid Beheshti University of Medical Sciences Tehran Iran; ^2^ Department of Medical Genetics, School of Medicine Tehran University of Medical Sciences Tehran Iran; ^3^ Nutrition & Metabolic Diseases Research Center Ahvaz Jundishapur University of Medical Sciences Ahvaz Iran; ^4^ Cancer Research Center Shahid Beheshti University of Medical Sciences Tehran Iran; ^5^ Genomic Research Center, Department of Medical Genetic Shahid Beheshti University of Medical Sciences Tehran Iran; ^6^ Master Student of Epidemiology Tehran University of Medical Sciences (TUMS) Tehran Iran; ^7^ Nurse Anesthesia Guilan University of Medical Sciences (GUMS) Langroud Iran; ^8^ Department of Anesthesiology Sabzevar University of Medical Sciences Sabzevar Iran; ^9^ Department of Clinical Nutrition, School of Nutrition and Food Sciences Shiraz University of Medical Sciences Shiraz Iran; ^10^ Department of Health Education and Promotion, Research Center of Health and Environment, School of Health Guilan University of Medical Sciences Rasht Iran; ^11^ Department of Clinical Nutrition and Dietetics, Faculty of Nutrition and Food Technology, National Nutrition and Food Technology Research Institute Shahid Beheshti University of Medical Sciences Tehran Iran; ^12^ Department of Pathology Firoozgar General Hospital, Iran University of Medical Sciences Tehran Iran; ^13^ Division of Endocrinology, Diabetes and Metabolism, Department of Medicine Cedars‐Sinai Medical Center California Los Angeles USA

**Keywords:** breast cancer, dietary intake, *FTO*, omega‐6 fatty acids, polymorphism

## Abstract

Breast cancer (BC) is the leading cause of cancer‐related deaths in females worldwide and is related to genetic and environmental factors. Dietary components may strongly influence the risk of BC. A possible association was also reported between the fat mass and obesity‐associated (*FTO*) single‐nucleotide polymorphisms (SNPs) and BC. This study aimed to investigate the impact of *FTO* rs9939609 polymorphism on the association between BC and dietary intake. This study was conducted on 180 women with BC as the case group and 360 healthy women as the control group. The dietary intakes were assessed by a valid 168‐item food frequency questionnaire (FFQ). The *FTO* gene was genotyped for rs9939609 polymorphism. After adjusting the confounding variables, there was no significant association between dietary intake and BC in individuals without risk allele. A positive association between dietary intake of omega‐6 fatty acids and BC was found only in individuals with risk allele of *FTO* gene (OR: 1.31, 95% CI: 1.08–1.60, *p*: 0.006). FTO gene risk allele may influence the effect of diet on breast cancer risk. Further studies are needed to assess the possible effects of the *FTO* genotype on the association between BC risk and dietary components.

## 1 BACKGROUND

Breast cancer (BC) is the most prevalent cancer and is the leading cause of cancer‐related deaths in females worldwide.^1^ It accounts for 1 million of about 10 million new neoplasms which are diagnosed every year worldwide.^2^ The prevalence of BC in Iranian women was reported to be 23.6%.^3^ An increase in the prevalence of BC is expected in the next decades along with the population ageing and exposure to cancer risk factors such as environmental carcinogens and unhealthy lifestyle.^1^, ^4^

Nutrition has a key role in cancer risk, and the evidence suggests that about 10%‐20 per cent of all cancer mortalities occurred due to malnutrition rather than underlying cancer.^5^, ^6^ Moreover, previous prospective studies indicated that there is an association between diets with low nutritional quality and an increased risk of developing chronic diseases, like cancer and obesity.^7^ For instance, some food and nutrients (e.g. saturated fats, red meat and processed meat) increase the circulating levels of endogenous estrogens, insulin‐like growth factors and pro‐inflammatory cytokines, thus supporting BC development. In contrast, polyunsaturated fatty acids, vitamins C and E, fresh fruits and vegetables were reported to have protective effects against BC onset or progression.^8^, ^9^ Hence, nutrition and dietary components can considerably influence the risk of cancer.^6^

On the contrary, BC is known as one of the most complicated diseases which is caused by a combination of environmental and genetic factors.^10^ Familial aggregation of BC was also frequently reported, which indicates the key role of genetics in the development of BC.^11^ Some genetic factors can influence the risk of BC. For example, several studies have documented that mutations in the *BRCA1* and *BRCA2* genes are responsible for hereditary BC, which accounts for 5%–10% of all BC.^11^, ^12^, ^13^ In addition, previous studies have revealed that dysfunction of some other genes including *Her2*, *C‐erbB‐2*, *c‐Myc*, *Cyclin, EGFR, IGF‐I and IGF‐II* can lead to the initiate BC.^11^, ^14^, ^15^

In addition, some genes such as fat mass and obesity‐associated (*FTO*) may have a dual effect on nutritional requirements and breast cancer risk. It has been established that the *FTO* gene has a role in appetite and food intake.^16^, ^17^ *FTO* gene is located on the chromosome region 16q12.2, and this gene is expressed ubiquitously with the highest expression in the liver, brain, hypothalamus and visceral fat.^18^ Previous studies have reported a strong relationship between *FTO* genotype and body mass index (BMI), and it has been demonstrated that *FTO* genotypes may affect the association of dietary macronutrients with body weight and BMI.^19^, ^20^, ^21^

On the contrary, recent studies reported that there is an association between variants of *FTO* including rs8050136, rs9939609, rs1477196, rs1121980, rs6499640, rs17817449, rs8047395, rs7206790 and rs11075995 polymorphisms and the risk of cancers.^22^ The association between the most typical *FTO* SNP (rs9939609) and several cancers such as renal, lung, breast, pancreas, endometrial and prostate cancer were frequently reported.^23^, ^24^, ^25^, ^26^ The frequency of FTO gene AA genotype of rs9939609 polymorphism in male and female subjects was reported to be 12.0 and 26.0%, respectively.^27^ Remarkably, it implies that the *FTO* variants may have an association with a broad range of diseases beyond their effect on BMI.^28^ For example, the association between *FTO* gene polymorphisms and BC was found to be associated with the status of oestrogen receptors and PI3 K/Akt signalling pathway.^29^ However, there is insufficient information to date to confirm the interplay of FTO gene and BC incidence and mortality.^30^ In addition, the *FTO* gene–nutrition interaction as an underlying mechanism of cancer has not been well established. Therefore, this study aimed to investigate the impact of *FTO* polymorphism on the association between BC and dietary intake in Iranian women.

## 2 METHODS

### 2.1 Study population and data collection

A case–control study was conducted on 180 patients with cancer as the case group and 360 healthy individuals as the control group. The required sample size was estimated based on the odds ratio (OR) of the previous studies.^31^ According to inclusion criteria, the participants were selected among women referred to the Cancer Research Center of Shohadaye Tajrish Hospital in Tehran, Iran. Individuals whose blood samples were collected at the beginning of the study were eligible to participate in the study according to the inclusion and exclusion criteria. For the case group, inclusion criteria were women with breast cancer, the age range of 35 to 70 years, consent of participation and recent cancer diagnosis within 3 months. For the control group, inclusion criteria were women with no malignancy, age range of 35 to 70 years and participation consent. Exclusion criteria were the inability to collect the required information and any disease that may affect the diet such as liver disease and diabetes. The case and control groups were matched by ethnicity (Persian). Basic information, including anthropometric measurements, medical history, physical activity (using the International Physical Activity Questionnaire), alcohol use, smoking, level of education and socio‐economic variables, was collected from the participants. Stadiometer was used for measuring the patient's height and weight was measured using a SECA Alpha 882 scale (SECA Corporation). Then, the patients' body mass index (BMI) was determined by dividing weight in kilograms by the square of height in metres (kg/m^2^).

### 2.2 Genotyping

Five millimetres (ml) of the blood samples of the participants was collected at the beginning of the study. The genomic deoxyribonucleic acid (DNA) was extracted from the blood samples using GeneAll DNA extraction kit (Incheon, Korea) following the manufacturer's instruction. Extracted DNA samples were amplified using PCR and master mix DNA polymerase (cat. No A180301; Ampliqon, Denmark). The FTO gene polymorphism rs9939609 was assessed using the tetra‐primer amplification refractory mutation system‐polymerase chain (Tetra‐ARMS PCR) technique using the PCR products. The sequences for the primers are presented in Supplementary file 1.

### 2.3 Dietary intake

The intake of macronutrients, micronutrients and calories was evaluated using a valid 168‐item FFQ consisted of 168 food items with standard portion sizes commonly consumed by Iranian people. Face‐to‐face interviews were administered by a trained dietitian. Data on food intake during the last year in the control group and related to food intake in the last year before cancer diagnosis in the case group were collected. All reported consumption frequencies were converted to grams per day by using household measures. Then, these data were used to determine macro and micronutrient intake by the Nutritionist IV software (version 7.0; N‐Squared Computing, Salem, OR, USA).

### 2.4 Statistical analysis

The participants in the two groups were compared in terms of demographic and pathological conditions by the independent t‐test and qi‐square test for quantitative and qualitative variables, respectively. The normal distribution of data was examined by the Kolmogorov–Smirnov test. The odds ratio of the association between BC and dietary components in people with different genotypes of the FTO gene was determined using logistic regression by adjusting the effect of confounding variables in different models: Model 1: adjusted for age, breast feeding duration, first menstruation age, post‐menopause age, breast cancer family history, number of pregnancies, smoking, using alcohol drinks and physical activity, and model 2: further adjustments for BMI. A dominant genetic model was used due to the low minor allele frequency (MAF) values. All analyses were performed using SPSS software (version 22.0; IBM Corp., Armonk, NY, USA) at a significance level of 0.05. The Hardy–Weinberg equation was used to calculate the allelic frequency in the studied population.

## 3 RESULTS

All variables were normally distributed. The average age of the cases and controls was 65 ± 27 and 68 ± 29 (*p* = 0.06), and the mean BMI of these groups was 29 ± 3.9 and 27 ± 2.8 kg/m^2^ (*p* < 0.01), respectively. The number of pregnancies and the rate of breastfeeding months were significantly lower, and the family history of BC was significantly higher in the case group compared with the control group (*p* = 0.01) (Table 1). Regarding the genotype of rs9939609 polymorphism of the *FTO* gene, no significant difference was found between the two groups in the dominant genetic model (AA and AT vs. TT) (*p* = 0.46). Based on the Hardy–Weinberg equation, allelic frequencies of A and T were 0.4 and 0.6, respectively. Also, there was no significant difference concerning the first menstrual age, menopausal age, smoking rate, alcohol consumption and physical activity between the groups.

**TABLE 1:** Characteristics and dietary intake of the participants

Variables	Cases (*n* = 180)	Controls (*n* = 360)	*p*
Age (y)	68 ± 29	65 ± 27	0.06
Height (cm)	156 ± 5	161 ± 6	0.01
Weight (kg)	71 ± 11	71 ± 10	0.86
BMI (kg/m^2^)	29.19 ± 3.91	27.27 ± 2.87	0.01
Breast feeding duration (month)	34 ± 29	59 ± 33	0.01
First menstruation Age (year)	13 ± 2	13 ± 2	0.51
Age of menopause (year)	47 ± 5	47 ± 5	0.89
Breast cancer family history (yes)	35%	14%	0.01
Number of pregnancies (*n*)	3 ± 2	4 ± 2	0.01
Smoking (yes)	2.9%	5%	0.34
Using alcohol drinks (yes)	98.5%	99.2%	0.62
Physical activity (hr/d)	2 ± 4.5	1.5 ± 1.5	0.51
*Genotype*			
TT	31%	35%	0.79
AT	58%	56%	
AA	11%	9%	
Calorie intake (Kcal/d)	2737 ± 925	2315 ± 1.066	0.01
Protein (g/d)	86.63 ± 41.572	84.93 ± 41.744	0.81
Carbohydrate (g/d)	402.1 ± 124	311.6 ± 170	<0.01
Total fat (g/d)	92.90 ± 42.277	93.34 ± 52.885	0.96
Cholesterol (mg/d)	2.420 ± 130	2.327 ± 161	0.71
Saturated fat (g/d)	2.929 ± 18.892	2.522 ± 13.006	0.12
MUFA (g/d)	3.049 ± 13.343	3.503 ± 24.546	0.19
PUFA (g/d)	1.941 ± 8.563	1.903 ± 12.364	0.83
Omega‐3 fatty acids (g/d)	1.253 ± 0.557	1.011 ± 1.033	0.09
Omega‐6 fatty acids (g/d)	5.452 ± 6.992	0.399 ± 0.586	<0.01
Sodium (mg/d)	5.661 ± 2.559	4.935 ± 4.288	0.23
Potassium (mg/d)	4.083 ± 1.829	4.635 ± 4.909	0.39
Vitamin A (RAE/d)	4.851 ± 265	8.767 ± 1.949	0.12
Beta carotene (μg/d)	3.116 ± 1.967	7.271 ± 2.316	0.16
Alpha carotene (μg/d)	5.435 ± 607	3.892 ± 3.048	0.32
Lutein (μg/d)	1.551 ± 794	2.385 ± 7.801	0.40
β‐Cryptoxanthin (μg/d)	2.973 ± 176	3.651 ± 495	0.30
Lycopene (μg/d)	7.620 ± 4.582	4.592 ± 9.664	0.02
Vitamin C (mg/d)	1.507 ± 113	2.179 ± 278	0.07
Calcium (mg/d)	1.277 ± 1.011	1.198 ± 674	0.57
Iron (mg/d)	19.78 ± 6.477	15.41 ± 12.131	0.01
Vitamin D (μg/d)	1.051 ± 0.842	1.794 ± 1.565	<0.01
Vitamin E (mg/d)	1.768 ± 11.446	1.746 ± 11.606	0.91
Alpha tocopherol (mg/d)	1.160 ± 7.650	1.286 ± 11.613	0.45
Thiamin (mg/d)	2.364 ± 0.962	1.555 ± 0.756	<0.01
Riboflavin (mg/d)	2.310 ± 1.372	2.183 ± 1.319	0.57
Niacin (mg/d)	2.431 ± 7.929	1.821 ± 9.212	<0.01
Vitamin B6 (mg/d)	1.823 ± 0.787	2.082 ± 1.188	0.13
Folate (μg/d)	6.735 ± 2.05	4.654 ± 3.08	<0.01
Vitamin B12 (μg/d)	3.933 ± 3.765	4.679 ± 3.138	0.19
Biotin (μg/d)	3.119 ± 14.816	3.198 ± 16.869	0.77
Pantothenic (mg/d)	5.311 ± 2.691	5.897 ± 2.547	0.18
Vitamin K (μg/d)	1.243 ± 55.014	3.995 ± 2.019	0.28
Phosphorus (mg/d)	1.450 ± 948	4.248 ± 3.275	0.32
Magnesium (mg/d)	3.822 ± 159	4.480 ± 379	0.20
Zinc (mg/d)	1.136 ± 6.041	1.280 ± 6.936	0.19
Copper (mg/d)	1.913 ± 0.606	1.838 ± 1.518	0.71
Manganese (mg/d)	5.772 ± 2.634	4.902 ± 3.724	0.12
Selenium (mg/d)	9.877 ± 40.823	8.259 ± 41.753	0.02
Fluoride (mg/d)	3.374 ± 1.882	5.286 ± 824	<0.01
Chromium (mg/d)	0.022 ± 0.089	0.071 ± 0.067	<0.01
Total fibre (g/d)	2.918 ± 11.318	3.232 ± 26.592	0.38
Soluble fibre (g/d)	1.055 ± 0.824	1.119 ± 2.316	0.83
Insoluble fibre (g/d)	5.421 ± 3.779	1.383 ± 1.062	0.32
Crude fibre (g/d)	1.139 ± 6.651	3.270 ± 42.434	<0.01
Sugar total (g/d)	1.430 ± 64.130	1.607 ± 92.727	0.19
Glucose (g/d)	2.150 ± 8.937	2.164 ± 15.959	0.95
Galactose (g/d)	4.293 ± 8.866	4.314 ± 3.317	0.99
Fructose (g/d)	2.711 ± 11.156	2.210 ± 16.627	0.04
Sucrose (g/d)	4.913 ± 27.130	5.899 ± 59.303	0.22
Lactose (g/d)	1.485 ± 24.062	1.632 ± 11.146	0.62
Maltose (g/d)	2.989 ± 1.551	1.024 ± 0.802	<0.01
Caffeine (mg/d)	1.876 ± 111	2.848 ± 49.043	<0.01

Abbreviations: BMI, body mass index; MUFA, monounsaturated fatty acids; PUFA, polyunsaturated fatty acids.

Regarding the dietary intake of the groups, the results showed that the intake of calories (2737 ± 925 vs. 2315 ± 1.066 kcal/d, *p =* 0.01), carbohydrates (402.1 ± 124 vs. 311.6 ± 170 g/d, *p* < 0.01), omega‐6 fatty acids (5.452 ± 6.992 vs. 0.399 ± 0.586 g/d, *p* < 0.01), iron (19.78 ± 6.477 vs. 1.541 ± 12.131 mg/d, *p =* 0.01), thiamine (2.364 ± 0.962 vs. 1.555 ± 0.756 mg/d, *p* < 0.01), niacin (2.431 ± 7.929 vs. 1.821 ± 9.212 mg/d, *p* < 0.01), folate (6.735 ± 2.05 vs. 4.654 ± 3.08 μg/d, *p* < 0.01) and maltose (2.989 ± 1.551 vs. 1.024 ± 0.802 g/d, *p* < 0.01) was higher in cancer patients compared with healthy individuals. On the contrary, the intake of vitamin D (1.794 ± 1.565 vs. 1.051 ± 0.842 μg/d, *p* < 0.01), fluoride (5.286 ± 824 vs. 3.374 ± 1.882 mg/d, *p* < 0.01), chromium (0.071 ± 0.067 vs. 0.022 ± 0.089 mg/d, *p* < 0.01), crude fibre (3.270 ± 42.434 vs. 1.139 ± 6.651 g/d, *p* < 0.01) and caffeine (2.848 ± 49.043 vs. 1.876 ± 111 mg/d, *p* < 0.01) in healthy individuals was higher than in patients with breast cancer (Table 1).

In terms of dietary intake between case and control groups with TT genotype of *FTO* rs9939609 polymorphism, results showed that intake of cholesterol (288.23 ± 137.27 vs. 176.12 ± 95.90 mg/d, *p* < 0.01), saturated fatty acids (29.61 ± 10.67 vs. 19.60 ± 6.50 g/d, *p* < 0.01), omega‐6 fatty acids (7.36 ± 5.80 vs. 1.42 ± 2.75 g/d, *p* < 0.01), vitamin B12 (4.94 ± 2.37 vs. 3.25 ± 1.80 μg/d, *p* = 0.01), phosphorus (200.97 ± 20.69 vs. 15.69 ± 87.64 mg/d, *p* < 0.01), fluoride (2736 ± 1604 vs. 479 ± 631 mg/d, *p* < 0.01), maltose (2.30 ± 1.26 vs. 1.09 ± 1.24 g/d, *p* = 0.01) and caffeine (180.17 ± 90.34 vs. 19.25 ± 36.11 mg/d, *p* < 0.01) in the case group was higher than in the control group (Table 2).

**TABLE 2:** Dietary intake between case and control groups with different genotypes

Variables	TT genotypes		*p*	AA/AT genotypes		*p*
	Cases (*N* = 56)	Controls (*n* = 126)		Cases (*n* = 124)	Controls (*n* = 234)	
Calorie intake (Kcal/d)	2589.57 ± 444.45	2261.98 ± 1146.99	0.39	2590.62 ± 507.86	2285.58 ± 1073.11	0.13
Protein (g/d)	84.78 ± 20.61	76.53 ± 43.22	0.56	83.38 ± 27.26	84.97 ± 44.93	0.84
Carbohydrate (g/d)	366.38 ± 70.89	337.47 ± 218.47	0.68	372.88 ± 63.24	301.67 ± 145.42	0.01
Total fat (g/d)	94.84 ± 20.15	94.43 ± 63.09	0.98	93.34 ± 24.07	91.40 ± 54.68	0.84
Cholesterol (mg/d)	288.23 ± 137.27	176.12 ± 95.90	<0.01	269.01 ± 141.72	225.15 ± 195.02	0.23
Saturated fat (g/d)	29.61 ± 10.67	19.60 ± 6.50	<0.01	29.67 ± 14.20	23.76 ± 12.66	0.02
MUFA (g/d)	34.60 ± 12.66	27.59 ± 14.81	0.17	33.25 ± 13.53	35.47 ± 28.84	0.67
PUFA (g/d)	20.43 ± 8.13	17.25 ± 9.89	0.34	18.96 ± 7.39	19.81 ± 13.01	0.72
Omega‐3 fatty acids (g/d)	1.33 ± 0.77	0.89 ± 0.59	0.05	1.24 ± 0.66	1.06 ± 1.11	0.39
Omega‐6 fatty acids (g/d)	7.36 ± 5.80	1.42 ± 2.75	<0.01	5.89 ± 5.45	0.88 ± 2.19	<0.01
Sodium (mg/d)	7077.65 ± 4645.62	6259.05 ± 3708.26	0.53	7418.36 ± 4202.02	5096.98 ± 4993.16	0.02
Potassium (mg/d)	5954.96 ± 4182.45	4500.51 ± 3640.31	0.28	5746.37 ± 3745.77	4976.15 ± 6619.09	0.53
Vitamin A (RAE/d)	681.41 ± 335.02	2351.18 ± 4773.37	0.29	657.98 ± 348.75	572.31 ± 379.46	0.25
Beta carotene (μg/d)	4750.26 ± 2726.77	25,909.97 ± 56,766.04	0.26	5010.22 ± 3046.66	3587.30 ± 3595.60	0.04
Alpha carotene (μg/d)	40,774.63 ± 50,738.50	274,498.71 ± 811,824.32	0.41	53,677.41 ± 61,803.93	3860.02 ± 19,669.38	<0.01
Lutein (μg/d)	1993.25 ± 1263.85	7510.44 ± 19,582.08	0.39	2156.89 ± 1294.91	1244.35 ± 968.15	<0.01
β‐Cryptoxanthin (μg /d)	467.35 ± 303.56	483.30 ± 592.36	0.93	441.01 ± 316.97	318.39 ± 359.36	0.08
Lycopene (μg/d)	9864.12 ± 6643.26	13,902.81 ± 22,504.58	0.60	9916.71 ± 7174.19	2293.30 ± 3512.05	<0.01
Vitamin C (mg/d)	196.20 ± 121.10	416.61 ± 554.12	0.24	195.09 ± 136.63	187.80 ± 185.54	0.83
Calcium (mg/d)	1239.57 ± 413.14	1275.67 ± 1212.01	0.92	1308.93 ± 710.95	1096.57 ± 541.63	0.05
Iron (mg/d)	18.63 ± 4.70	21.69 ± 23.96	0.69	18.18 ± 4.49	14.62 ± 8.21	0.02
Vitamin D (μg/d)	2.04 ± 1.43	1.40 ± 1.39	0.21	1.91 ± 1.27	1.77 ± 1.50	0.63
Vitamin E (mg/d)	19.17 ± 9.69	21.32 ± 22.47	0.77	16.81 ± 8.75	17.23 ± 8.22	0.79
Alpha tocopherol (mg/d)	13.02 ± 6.22	17.28 ± 24.98	0.60	11.66 ± 5.78	12.22 ± 7.18	0.68
Thiamin (mg/d)	2.07 ± 0.58	1.60 ± 0.91	0.14	2.13 ± 0.67	1.62 ± 0.85	<0.01
Riboflavin (mg/d)	2.27 ± 0.65	2.60 ± 2.90	0.72	2.30 ± 0.94	1.99 ± 0.75	0.04
Niacin (mg/d)	21.48 ± 6.74	18.07 ± 14.35	0.47	21.74 ± 7.88	19.56 ± 8.94	0.20
Vitamin B6 (mg/d)	1.82 ± 0.53	2.76 ± 2.48	0.26	1.91 ± 0.63	1.87 ± 0.68	0.71
Folate total (g/d)	596.88 ± 179.82	514.82 ± 405.19	0.54	614.36 ± 155.08	480.06 ± 346.32	0.04
Folate DFE (μg/d)	699.65 ± 230.28	568.51 ± 451.45	0.38	721.58 ± 213.84	493.01 ± 371.46	<0.01
Vitamin B12 (μg/d)	4.94 ± 2.37	3.25 ± 1.80	0.01	4.40 ± 3.32	4.67 ± 3.27	0.67
Biotin (μg/d)	30.69 ± 11.64	38.80 ± 27.92	0.38	31.76 ± 12.95	29.34 ± 11.65	0.29
Pantothenic (mg/d)	5.51 ± 1.61	5.58 ± 3.54	0.94	5.61 ± 1.91	5.81 ± 2.41	0.65
Vitamin K (μg/d)	306.17 ± 265.19	1757.50 ± 5096.46	0.39	312.55 ± 231.58	132.62 ± 151.62	<0.01
Phosphorus (mg/d)	200.97 ± 20.69	15.69 ± 87.64	<0.01	155,502.93 ± 188,116.67	17,395.97 ± 86,793.14	<0.01
Magnesium (mg/d)	404.39 ± 108.80	614.56 ± 818.89	0.43	413.93 ± 110.34	404.66 ± 238.94	0.83
Zinc (mg/d)	12.20 ± 4.44	12.59 ± 7.73	0.87	11.74 ± 4.64	12.59 ± 6.48	0.48
Copper (mg/d)	1.92 ± 0.51	2.38 ± 2.94	0.63	1.83 ± 0.53	1.73 ± 1.18	0.63
Manganese (mg/d)	5.73 ± 1.67	6.29 ± 7.23	0.81	5.78 ± 1.78	4.96 ± 3.16	0.16
Selenium (mg/d)	99.44 ± 31.87	82.07 ± 49.09	0.29	98.48 ± 34.53	87.68 ± 50.12	0.25
Fluoride (mg/d)	2.74 ± 1604	4.79 ± 631	<0.01	3.09.80 ± 1800.11	4.79 ± 614.69	<0.01
Chromium (mg/d)	0.06 ± 0.06	0.08 ± 0.11	0.66	0.07 ± 0.07	0.08 ± 0.08	0.55
Total Fibre (g/d)	30.20 ± 14.24	40.40 ± 44.04	0.48	31.24 ± 13.87	32.28 ± 26.25	0.83
Soluble fibre (g/d)	1.93 ± 1.49	1.14 ± 1.36	0.11	1.99 ± 1.52	0.8 ± 0.69	<0.01
Insoluble fibre (g/d)	584.13 ± 747.25	951.86 ± 2832.80	0.70	623.26 ± 746.69	13.02 ± 44.43	<0.01
Crude fibre (g/d)	29.81 ± 26.56	30.29 ± 30.98	0.96	35.17 ± 29.88	40.76 ± 55.09	0.60
Sugar total (g/d)	145.23 ± 50.55	159.73 ± 105.80	0.67	148.96 ± 47.35	155.23 ± 80.62	0.67
Glucose (g/d)	19.56 ± 10.05	24.59 ± 20.94	0.47	21.35 ± 10.78	21.13 ± 16.10	0.94
Galactose (g/d)	4.41 ± 3.02	3.07 ± 2.24	0.12	5.11 ± 6.63	4.38 ± 3.96	0.41
Fructose (g/d)	23.43 ± 11.64	26.29 ± 23.50	0.71	24.83 ± 12.55	21.51 ± 16.16	0.28
Sucrose (g/d)	57.20 ± 37.54	49.45 ± 59.31	0.66	55.53 ± 31.11	60.08 ± 51.82	0.63
Lactose (g/d)	16.38 ± 9.72	12.20 ± 11.20	0.30	16.87 ± 17.86	16.48 ± 10.92	0.87
Maltose (g/d)	2.30 ± 1.26	1.09 ± 1.24	0.01	2.34 ± 1.34	1.25 ± 0.93	<0.01
Caffeine (mg/d)	180.17 ± 90.34	19.25 ± 36.11	<0.01	184.33 ± 99.20	23.17 ± 36.34	<0.01

Abbreviations: MUFA, monounsaturated fatty acids, PUFA, polyunsaturated fatty acids.

Regarding dietary intake between case and control groups with AA/AT genotype of *FTO* rs9939609 polymorphism, results showed that intake of carbohydrates (372 ± 63 vs. 301 ± 145 g/d, *p* = 0.01), saturated fat (29.67 ± 14.20 vs. 23.76 ± 12.66 g/d, *p* = 0.02), omega‐6 (5.89 ± 5.45 vs. 0.88 ± 2.19 g/d, *p* < 0.01), sodium (7418 ± 4202 vs. 5096 ± 4993 mg/d, *p* = 0.02), beta‐carotene (5010 ± 3046 vs.3587 ± 3595 μg/d, *p* = 0.04), alpha‐carotene (53,677 ± 61,803 vs. 3860 ± 1966 μg/d, *p* < 0.01), lutein (2156 ± 1249 vs. 1244 ± 968 μg/d, *p* < 0.01), lycopene (9916 ± 7174, vs. 2293 ± 3512 μg/d, *p* < 0.01), iron (18.18 ± 4.49 vs. 14.62 ± 8.21 mg/d, *p* = 0.02), thiamin (2.13 ± 0.67 vs. 1.62 ± 0.85 mg/d, *p* < 0.01), riboflavin (2.30 ± 0.94 vs. 1.99 ± 0.75 mg/d, *p* = 0.04), folate (614 ± 155 vs. 480 ± 346 μg/d, *p* = 0.04), vitamin K (312 ± 231 vs. 132 ± 151 μg/d, *p* < 0.01), phosphorus (155 ± 188 vs. 173 ± 867 mg/d, *p* < 0.01), fluoride (3087 ± 1800 vs. 479 ± 614 μg/d, *p* < 0.01), soluble fibre (1.99 ± 1.52 vs. 0.8 ± 0.69 g/d, *p* < 0.01), insoluble fibre (623 ± 746 vs. 13 ± 44 g/d, *p* < 0.01), maltose (2.34 ± 1.34 vs. 1.25 ± 0.93 g/d, *p* < 0.01) and caffeine (184 ± 99 vs. 23 ± 36 mg/d, *p* < 0.01) was higher in case of the group compared with the control group (Table 2).

After considering the *FTO* genotype differences between the groups, the results indicated that the amount of nutrients intake in individuals with one or two risk alleles in each of the two groups was not significantly different from those without risk alleles (Table 3).

**TABLE 3:** Comparison of food intake in individuals with polymorphism with those without polymorphism in case and control groups

Variables	Cases (*n* = 180)		*p*	Controls (*n* = 360)		*p*
	TT	AA & AT		TT	AA & AT	
Calorie intake (Kcal/d)	2743 ± 922	2792 ± 1.064	0.85	2258 ± 1.216	2263 ± 1.106	0.99
Protein (g/d)	82.0 ± 34.626	88.47 ± 51.611	0.83	7951 ± 44.743	8521 ± 46.091	0.75
Carbohydrate (g/d)	397.0 ± 144	411.8 ± 125	0.68	336.9 ± 231	296.9 ± 149	0.64
Total fat (g/d)	96.81 ± 41.034	93.47 ± 49.936	0.77	95.66 ± 66.801	90.93 ± 56.571	0.85
Cholesterol (mg/d)	261.6 ± 125	234.1 ± 144	0.43	192.0 ± 86.532	230.1 ± 200	0.43
Saturated fat (g/d)	29.52 ± 13.553	30.14 ± 24.541	0.90	18.81 ± 6.372	23.65 ± 12.953	0.14
MUFA (g/d)	3154 ± 13.117	3074 ± 15.298	0.83	2918 ± 14.774	3583 ± 29.730	0.38
PUFA (g/d)	2142 ± 10.072	1873 ± 8.515	0.29	1615 ± 9.829	1990 ± 13.293	0.37
Omega‐3 fatty acids (g/d)	1397 ± 0.698	1213 ± 0.530	0.28	0811 ± 0.570	1047 ± 1.127	0.41
Omega‐6 fatty acids (g/d)	6486 ± 7.931	5450 ± 7.519	0.61	0602 ± 0.944	0331 ± 0.410	0.42
Sodium (mg/d)	5237 ± 2.333	5610 ± 2.390	0.55	5615 ± 3.286	4682 ± 4.871	0.52
Potassium (mg/d)	4271 ± 1.890	4114 ± 2.121	0.76	4500 ± 3.640	5052 ± 6.841	0.76
Vitamin A (RAE/d)	5291 ± 248	4823 ± 308	0.51	2550 ± 5.018	5591 ± 379	0.27
Beta carotene (μg/d)	3154 ± 1.751	3265 ± 2.373	0.83	2826 ± 5.968	3261 ± 3.176	0.24
Alpha carotene (μg/d)	5190 ± 521	6219 ± 745	0.52	2744 ± 8.118	2902 ± 387	0.34
Lutein (μg/d)	1426 ± 578	1644 ± 913	0.25	8115 ± 2.067	1210 ± 888	0.35
β‐Cryptoxanthin (μg/d)	3034 ± 187	2923 ± 179	0.82	4885 ± 628	3045 ± 363	0.42
Lycopene (μg/d)	7997 ± 4.444	6806 ± 4.261	0.30	1390 ± 2.250	1977 ± 3.109	0.15
Vitamin C (mg/d)	1789 ± 82.964	1485 ± 146	0.29	4314 ± 585	1805 ± 184	0.24
Calcium (mg/d)	1265 ± 612	1357 ± 1.361	0.71	1349 ± 1.261	1122 ± 538	0.61
Iron (mg/d)	1931 ± 6.970	1980 ± 6.262	0.78	2147 ± 25.410	1426 ± 8.269	0.43
Vitamin D (μg/d)	1331 ± 1.015	0994 ± 0.787	0.18	1400 ± 1.391	1837 ± 1.534	0.44
Vitamin E (mg/d)	1966 ± 12.848	1753 ± 12.312	0.52	2117 ± 23.831	1675 ± 8.276	0. 60
Alpha tocopherol (mg/d)	1308 ± 8.649	1130 ± 8.132	0.42	1795 ± 26.405	1203 ± 7.386	0.52
Thiamin (mg/d)	2233 ± 0.873	2463 ± 1.086	0.36	1489 ± 0.884	1596 ± 0.868	0.76
Riboflavin (mg/d)	2317 ± 1.064	2413 ± 1.752	0.79	2701 ± 3.066	2011 ± 0.766	0.52
Niacin (mg/d)	2297 ± 7.712	2481 ± 7.958	0.37	1701 ± 14.802	1915 ± 9.067	0.69
Vitamin B6 (mg/d)	1820 ± 0.652	1888 ± 0.970	0.74	2871 ± 2.608	1829 ± 0.680	0.27
Folate total (g/d)	6721 ± 244	6824 ± 197	0.86	5158 ± 429	4766 ± 358	0.81
Folate DFE (μg/d)	8056 ± 299	8341 ± 291	0.72	5642 ± 478	4848 ± 383	0.66
Vitamin B12 (μg/d)	4078 ± 1.999	4188 ± 5.095	0.90	3238 ± 1.914	4688 ± 3.336	0.12
Biotin (μg/d)	3335 ± 12.539	3077 ± 17.258	0.50	3943 ± 29.547	2891 ± 11.703	0.33
Pantothenic (mg/d)	5359 ± 2.298	5415 ± 3.327	0.94	5701 ± 3.745	5810 ± 2.504	0.94
Vitamin K (μg/d)	1268 ± 50.526	1264 ± 60.966	0.98	1926 ± 5.375	1258 ± 149	0.34
Phosphorus (mg/d)	1424 ± 646	1521 ± 1.247	0.68	1500 ± 877	1550 ± 742	0.88
Magnesium (mg/d)	3796 ± 162	3840 ± 167	0.92	6266 ± 867	4110 ± 245	0.48
Zinc (mg/d)	1109 ± 5.806	1141 ± 6.619	0.84	1205 ± 8.008	1236 ± 6.566	0.92
Copper (mg/d)	1942 ± 0.648	1896 ± 0.598	0.78	2385 ± 3.125	1780 ± 1.207	0.58
Manganese (mg/d)	5323 ± 1.796	5810 ± 1.917	0.32	5779 ± 7.472	4941 ± 3.237	0.75
Selenium (mg/d)	9151 ± 33.884	1019 ± 38.551	0.27	7071 ± 35.478	8576 ± 51.306	0.33
Fluoride (mg/d)	3313 ± 1.549	3272 ± 1.675	0.92	4795 ± 631	4.283 ± 539	0.83
Chromium (mg/d)	0003 ± 0.007	0.026 ± 0.074	0.06	0049 ± 0.041	0.083 ± 0.084	0.13
Total Fibre (g/d)	3150 ± 14.805	2896 ± 9.109	0.46	4079 ± 46.697	3265 ± 26.468	0.63
Soluble fibre (g/d)	1204 ± 1.174	1045 ± 0.648	0.55	1265 ± 1.390	0784 ± 0.673	0.34
Insoluble fibre (g/d)	5855 ± 4.914	5600 ± 3.467	0.83	9518 ± 2.832	4975 ± 5.471	0.35
Crude fibre (g/d)	1153 ± 6.458	1142 ± 6.039	0.95	3029 ± 30.988	4076 ± 55.091	0.48
Sugar total (g/d)	1458 ± 73.343	1469 ± 65	0.95	1622 ± 111	1538 ± 83.192	0.84
Glucose (g/d)	2189 ± 9.356	21,924 ± 9.857	0.99	2422 ± 22.183	2129 ± 16.623	0.72
Galactose (g/d)	4019 ± 3.233	4986 ± 12.405	0.64	3075 ± 2.249	4428 ± 4.024	0.21
Fructose (g/d)	2811 ± 12.866	2722 ± 11.563	0.79	2723 ± 24.730	2168 ± 16.588	0.54
Sucrose (g/d)	5311 ± 34.881	4777 ± 23.931	0.52	5371 ± 54.719	5817 ± 52.808	0.83
Lactose (g/d)	1528 ± 10.686	1647 ± 33.355	0.84	1220 ± 11.208	1650 ± 11.117	0.33
Maltose (g/d)	2827 ± 1.478	3062 ± 1.707	0.57	0734 ± 0.538	1199 ± 0.926	0.07
Caffeine (mg/d)	1801 ± 90.340	1843 ± 99.203	0.87	1925 ± 36.116	2317 ± 36.344	0.78

*Note*: Adjusted for age, BMI, breast feeding duration, first menstruation age, post‐menopause age, breast cancer family history, number of pregnancies, smoking, using alcohol drinks and physical activity.

Abbreviations: MUFA, monounsaturated fatty acids, PUFA, polyunsaturated fatty acids.

The results of logistic regression of the association between BC and dietary intake regardless of *FTO* genotypes showed that there was a significant association between dietary intake of calorie (OR: 1.01, 95% CI: 1.00–1.02, *p*: 0.04) and omega‐6 fatty acids (OR: 1.25, 95% CI: 1.14–1.38, *p* < 0.01) (Table 4). Moreover, the results of logistic regression of the association between BC and dietary intake based on *FTO* genotype showed that there was no significant association between dietary intake and BC in individuals without risk allele after adjusting confounding variables including age, breastfeeding duration, first menstruation age, post‐menopause age, breast cancer family history, number of pregnancies, smoking, using alcohol drinks and physical activity (Model 1). Further adjustment for BMI did not change the results (Model 2) (Table 5).

**TABLE 4:** Logistic regression of the association between breast cancer and dietary intake

Variables	OR (CI 95%)	*p*
Calorie intake	1.01 (1–1.02)	0.04
Protein	0.94 (0.89–1.00)	0.05
Carbohydrate	0.97 (0.93–1.00)	0.24
Total fat	0.91 (0.83–1.00)	0.06
Cholesterol	1.00 (0.99–1.00)	0.65
Saturated fat	0.98 (0.93–1.00)	0.60
MUFA	0.99 (0.95–1.00)	0.76
PUFA	0.99 (0.91–1.00)	0.82
Omega‐3 fatty acids	1.38 (0.68–2.81)	0.37
Omega‐6 fatty acids	1.25 (1.14–1.38)	<0.01
Sodium	1.00 (1.00–1.00)	0.82
Potassium	1.00 (1.00–1.00)	0.53
Vitamin A	0.99 (0.99–1.00)	0.45
Beta carotene	1.00 (1.00–1.00)	0.43
Alpha carotene	1.00 (1.00–1.00)	0.54
Lutein	1.00 (1.00–1.00)	0.38
β‐Cryptoxanthin	0.99 (0.99–1.00)	0.49
Lycopene	1.00 (1.00–1.00)	0.16
Vitamin C	1.00 (0.99–1.00)	0.77
Calcium	1.00 (0.99–1.00)	0.88
Iron	0.96 (0.80–1.15)	0.68
Vitamin D	1.17 (0.85–1.59)	0.32
Vitamin E	0.90 (0.80–1.02)	0.10
Alpha tocopherol	1.00 (0.84–1.19)	0.95
Thiamin	1.19 (0.22–6.27)	0.83
Riboflavin	1.01 (0.29–3.45)	0.98
Niacin	1.01 (0.94–1.08)	0.76
Vitamin B6	0.90 (0.32–2.50)	0.84
Folate total	1.00 (0.99–1.01)	0.73
Folate DFE	0.99 (0.99–1.00)	0.62
Vitamin B12	1.01 (0.83–1.22)	0.90
Biotin	1.03 (0.98–1.07)	0.18
Pantothenic	0.80 (0.54–1.20)	0.28
Vitamin K	1.00 (0.99–1.00)	0.96
Phosphorus	1.00 (1.00–1.00)	0.11
Magnesium	0.99 (0.98–1.00)	0.66
Zinc	0.97 (0.83–1.12)	0.69
Copper	1.53 (0.34–6.84)	0.57
Manganese	0.75 (0.49–1.13)	0.17
Selenium	1.00 (0.97–1.02)	0.99
Fluoride	1.00 (1.00–1.01)	0.75
Chromium	16.17 (0.01–27)	0.46
Total fibre	1.01 (0.95–1.07)	0.64
Soluble fibre	1.29 (0.99–1.69)	0.05
Insoluble fibre	1.00 (0.99–1.00)	0.98
Crude fibre	1.00 (0.98–1.02)	0.68
Sugar total	0.99 (0.97–1.00)	0.20
Glucose	0.99 (0.95–1.03)	0.71
Galactose	1.02 (0.90–1.16)	0.68

Abbreviations: MUFA, monounsaturated fatty acids, PUFA, polyunsaturated fatty acids.

**TABLE 5:** Logistic regression of the association between breast cancer and dietary intake in individuals with (AA/AT) and without risk allele (TT) of rs9939609 FTO gene polymorphism

Variables	TT genotype				AA/AT genotype			
	Model 1		Model 2		Model 1		Model 2	
	OR (CI 95%)	*p*	OR (CI 95%)	*p*	OR (CI 95%)	*p*	OR (CI 95%)	*p*
Calorie intake	1.01 (0.96–1.05)	0.66	0.99 (0.94–1.05)	0.91	1.01 (0.99–1.03)	0.25	1.01 (0.98–1.03)	0.34
Protein	0.94 (0.72–1.21)	0.63	0.96 (0.70–1.33)	0.84	0.88 (0.77–1.01)	0.07	0.86 (0.74–1.01)	0.08
Carbohydrate	0.97 (0.82–1.15)	0.77	1.01 (0.81–1.27)	0.87	0.97 (0.89–1.06)	0.57	0.99 (0.89–1.10)	0.86
Total fat	0.90 (0.60–1.35)	0.62	0.98 (0.58–1.66)	0.95	0.91 (0.75–1.11)	0.39	0.92 (0.72–1.18)	0.55
Cholesterol	0.99 (0.98–1.01)	0.51	0.99 (0.97–1.01)	0.41	1.00 (0.99–1.00)	0.14	1.00 (0.99–1.01)	0.07
Saturated fat	0.92 (0.73–1.15)	0.47	0.95 (0.72–1.25)	0.74	0.95 (0.84–1.08)	0.47	0.89 (0.76–1.04)	0.15
MUFA	0.99 (0.83–1.19)	0.98	0.97 (0.76–1.25)	0.86	0.99 (0.90–1.08)	0.89	0.97 (0.87–1.09)	0.71
PUFA	1.08 (0.73–1.59)	0.69	1.17 (0.71–1.92)	0.52	1.03 (0.86–1.23)	0.72	0.93 (0.74–1.17)	0.57
Omega‐3 fatty acids	3.68 (0.11–118/6)	0.46	2.64 (0.02–269‐89)	0.68	0.68 (0.13–3.51)	0.64	1.05 (0.12–8.95)	0.95
Omega‐6 fatty acids	1.40 (0.89–2.20)	0.14	1.39 (0.85–2.27)	0.18	1.31 (1.08–1.60)	<0.01	1.26 (1.00–1.59)	0.04
Sodium	1.00 (1.00–1.00)	0.82	1.00 (1.00–1.00)	0.86	1.00 (1.00–1.00)	0.27	1.00 (1.00–1.00)	0.51
Potassium	1.00 (1.00–1.00)	0.52	1.00 (1.00–1.00)	0.28	1.00 (1.00–1.00)	0.97	1.00 (1.00–1.00)	0.40
Vitamin A	0.99 (0.99–1.00)	0.85	0.99 (0.98–1.00)	0.87	1.00 (0.99–1.00)	0.92	1.00 (0.99–1.00)	0.91
Beta carotene	0.99 (0.99–1.00)	0.26	0.99 (0.99–1.00)	0.33	1.00 (0.99–1.00)	0.28	1.00 (0.99–1.00)	0.13
Alpha carotene	1.00 (1.00–1.00)	0.90	1.00 (1.00–1.00)	0.91	1.00 (1.00–1.00)	0.64	1.00 (1.00–1.00)	0.43
Lutein	1.00 (0.99–1.00)	0.25	1.00 (0.99–1.00)	0.21	1.00 (1.00–1.00)	0.26	1.00 (0.99–1.00)	0.59
β‐Cryptoxanthin	1.00 (0.99–1.00)	0.94	1.00 (0.99–1.00)	0.90	0.99 (0.99–1.00)	0.25	0.99 (0.99–1.00)	0.64
Lycopene	1.00 (1.00–1.00)	0.82	1.00 (1.00–1.00)	0.65	1.00 (1.00–1.00)	0.18	1.00 (1.00–1.00)	0.22
Vitamin C	1.00 (0.98–1.01)	0.96	0.99 (0.97–1.01)	0.61	0.99 (0.99–1.00)	0.76	0.99 (0.99–1.00)	0.58
Calcium	0.99 (0.98–1.00)	0.55	0.99 (0.98–1.00)	0.54	1.00 (0.99–1.00)	0.64	1.00 (0.99–1.00)	0.62
Iron	1.46 (0.56–3.82)	0.43	1.52 (0.40–5.83)	0.53	0.97 (0.64–1.47)	0.90	0.96 (0.57–1.61)	0.85
Vitamin D	1.10 (0.22–4.76)	0.89	1.51 (0.23–9.98)	0.66	1.53 (0.75–3.11)	0.24	1.31 (0.54–3.20)	0.54
Vitamin E	0.81 (0.48–1.38)	0.45	0.91 (0.49–1.70)	0.78	0.89 (0.67–1.18)	0.44	0.92 (0.66–1.28)	0.63
Alpha tocopherol	1.14 (0.56–2.33)	0.70	1.01 (0.38–2.65)	0.98	0.91 (0.60–1.39)	0.68	0.95 (0.57–1.57)	0.85
Thiamin	4.42 (0–76)	0.69	7.86 (0–55)	0.64	2.87 (0.06–118)	0.57	2.74 (0.01–409)	0.69
Riboflavin	0.56 (0–385.93)	0.86	30.44 (0–38)	0.47	0.71 (0.05–10.10)	0.80	1.10 (0.04–30.21)	0.95
Niacin	0.85 (0.61–1.18)	0.33	0.79 (0.52–1.18)	0.25	1.04 (0.89–1.22)	0.56	1.10 (0.89–1.35)	0.37
Vitamin B6	0.06 (0–3.34)	0.17	0.02 (0–3.80)	0.15	1.57 (0.19–12.58)	0.67	1.27 (0.09–16.57)	0.85
Folate total	1.01 (0.96–1.06)	0.59	1.00 (0.94–1.06)	0.95	1.00 (0.98–1.03)	0.50	1.02 (0.99–1.05)	0.12
Folate	0.98 (0.95–1.02)	0.52	0.99 (0.94–1.04)	0.80	0.99 (0.97–1.00)	0.45	0.98 (0.95–1.00)	0.13
Vitamin B12	1.67 (0.63–4.43)	0.29	2.05 (0.57–7.40)	0.27	0.90 (0.64–1.28)	0.58	0.77 (0.51–1.16)	0.21
Biotin	1.11 (0.93–1.31)	0.23	1.16 (0.93–1.44)	0.17	1.05 (0.95–1.15)	0.30	1.03 (0.92–1.15)	0.58
Pantothenic	2.37 (0.22–24.95)	0.47	1.74 (0.07–39.46)	0.72	0.64 (0.25–1.64)	0.36	0.55 (0.17–1.74)	0.31
Vitamin K	1.00 (0.99–1.01)	0.52	1.00 (0.98–1.01)	0.82	0.99 (0.99–1.00)	0.62	0.99 (0.99–1.00)	0.82
Phosphorus	1.00 (1.00–1.00)	0.07	1.00 (1.00–1.00)	0.09	1.00 (1.00–1.00)	0.39	1.00 (1.00–1.00)	0.22
Magnesium	0.98 (0.94–1.03)	0.56	0.97 (0.91–1.04)	0.43	0.99 (0.97–1.01)	0.56	0.99 (0.96–1.01)	0.56
Zinc	1.12 (0.54–2.29)	0.75	1.64 (0.53–5.01)	0.38	0.98 (0.69–1.41)	0.94	1.06 (0.68–1.66)	0.77
Copper	0.73 (0–434.51)	0.92	1.50 (0–45)	0.92	2.64 (0.10–65)	0.55	3.11 (0.06–162)	0.57
Manganese	0.64 (0.09–4.25)	0.64	1.34 (0.09–19.37)	0.83	0.55 (0.23–1.31)	0.18	0.52 (0.17–1.63)	0.26
Selenium	0.99 (0.86–1.14)	0.93	0.93 (0.77–1.12)	0.47	0.99 (0.94–1.03)	0.70	0.96 (0.89–1.02)	0.25
Fluoride	1.00 (1.00–1.00)	0.18	1.00 (0.99–1.00)	0.31	1.00 (1.00–1.00)	0.60	1.00 (1.00–1.00)	0.30
Chromium	0 (0–46)	0.44	0 (0–87)	0.37	97 (0–50)	0.24	88 (0.22–35)	0.06
Total Fibre	1.00 (0.82–1.23)	0.96	1.11 (0.85–1.44)	0.43	1.02 (0.90–1.16)	0.67	0.99 (0.85–1.15)	0.93
Soluble fibre	2.02 (0.59–6.92)	0.25	2.77 (0.49–15.61)	0.24	1.55 (0.66–3.60)	0.30	1.81 (0.61–5.33)	0.28
Insoluble fibre	1.00 (0.99–1.00)	0.84	0.99 (0.99–1.00)	0.71	1.00 (0.99–1.00)	0.72	1.00 (0.99–1.00)	0.77
Crude fibre	0.95 (0.86–1.05)	0.39	0.95 (0.84–1.08)	0.46	1.00 (0.96–1.03)	0.99	0.99 (0.95–1.03)	0.82
Sugar total	1.00 (0.93–1.08)	0.87	0.99 (0.91–1.09)	0.98	0.99 (0.95–1.02)	0.69	0.97 (0.93–1.02)	0.33
Glucose	0.88 (0.75–1.04)	0.15	0.84 (0.68–1.04)	0.11	1.01 (0.92–1.10)	0.68	1.01 (0.91–1.13)	0.77
Galactose	1.00 (0.39–2.54)	0.99	0.70 (0.20–2.46)	0.58	1.10 (0.85–1.42)	0.44	1.202 (0.89–1.61)	0.22

*Note*: Model 1: Adjusted for age, breast feeding duration, first menstruation age, post‐menopause age, breast cancer family history, number of pregnancies, smoking, using alcohol drinks and physical activity. Model 2: further adjustments for BMI.

Abbreviations: MUFA, monounsaturated fatty acids; PUFA, polyunsaturated fatty acids.

**FIGURE 1:**
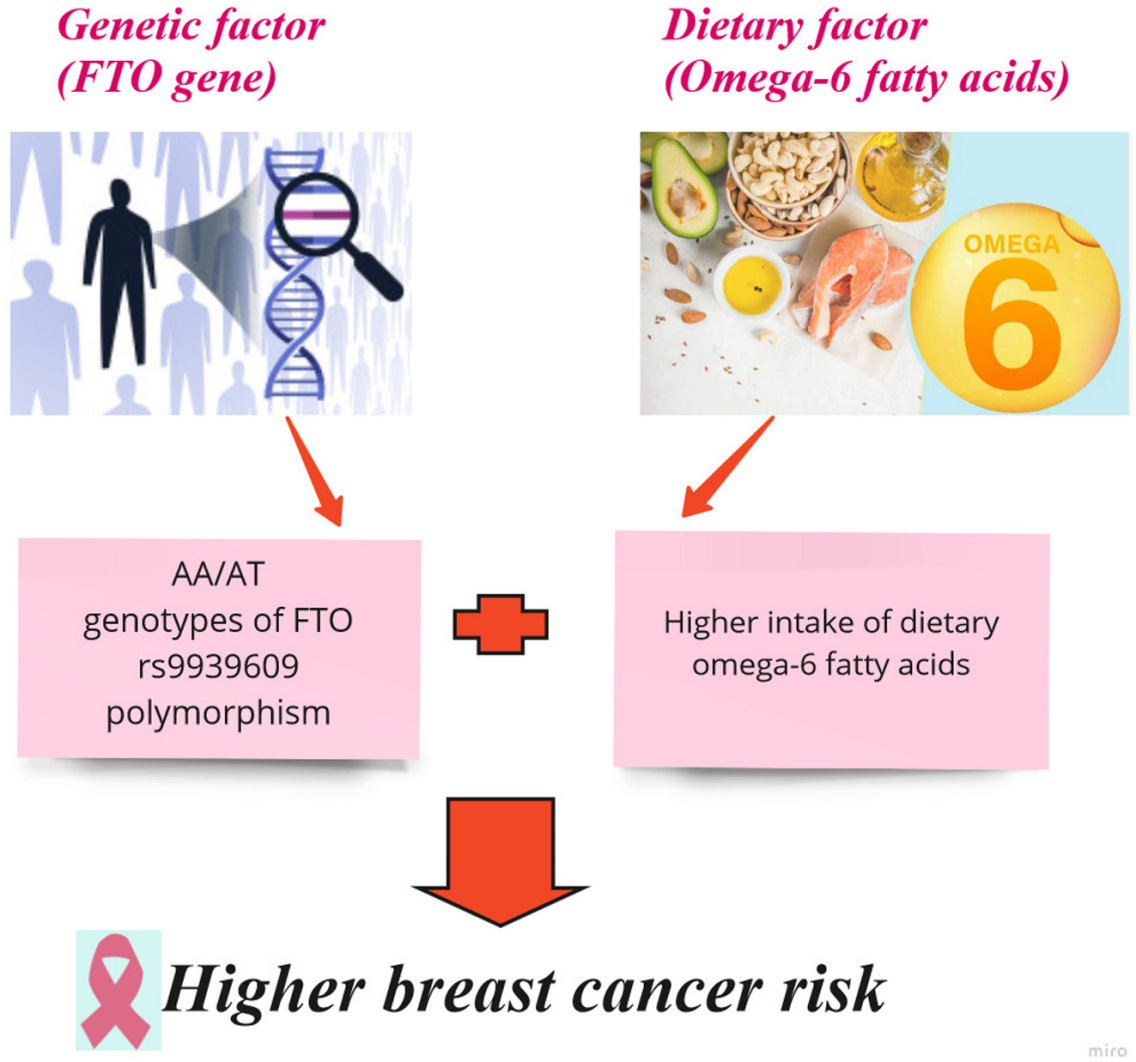
Association between breast cancer and dietary intake of omega‐6 fatty acids in individuals with risk allele (AA/AT) of 9939609 FTO gene polymorphism

In individuals with the *FTO* risk allele (AA/AT genotype), the results of logistic regression showed a significant association between BC with dietary intake of omega‐6 fatty acids (OR: 1.31, 95% CI: 1.08–1.60, *p*: <0.01) after adjusting for confounding factors such as age, breastfeeding duration, first menstruation age, post‐menopause age, breast cancer family history, number of pregnancies, smoking, using alcohol drinks and physical activity (Figure 1). Further adjustment for BMI did not change the results (OR: 1.26, 95%CI: 1.00–1.59, *p*: 0.04 for PUFA‐6 and OR: 1.00, 95%CI: 1.00–1.00, *p*: 0.03 for F) (Table 5).

## 4 DISCUSSION

The results of this case–control study indicated that the intake of calories, carbohydrates, omega‐6 fatty acids, iron, thiamine, niacin, folate and maltose was higher in BC patients compared with healthy individuals, while the intake of vitamin D, fluoride, chromium, crude fibre and caffeine in healthy individuals was higher than in patients with BC. The most interesting finding was a significant positive association between dietary intake of omega‐6 fatty acids and BC only in individuals with risk allele of *FTO* rs9939609 polymorphism. The result on the association between omega‐6 fatty acids and BC was in line with the previous studies that have reported the positive association between omega‐6 fatty acids and BC risk.^32^, ^33^, ^34^ However, Peppone et al. in a multicentre randomized controlled trial reported that omega‐6 fatty acids supplementation significantly decreased pro‐inflammatory markers in the TNF‐α signalling pathway among BC survivors.^35^ There is evidence indicating that omega‐6 fatty acids (ω‐6 PUFA) can be involved in pro‐cancer processes. For example, it was reported that ω‐6 PUFA increases the proliferation of the breast carcinoma cell line BT‐474.^36^ The pro‐tumour mechanisms of ω‐6 PUFA can be explained by the production of some reactive species, which can act on macromolecules causing DNA damage. In addition, lipids may alter the structure of chromatin and thus affect the accessibility of carcinogens to some specific genes, DNA repair factors and transcription complexes.^37^ Moreover, angiogenesis, the key process for tumour growth and metastasis, occurs through the synthesis of omega‐6 fatty acids.^34^ Several omega‐6 fatty acids such as prostaglandins, leukotrienes, thromboxanes and hydroxyeicosatraenoic acids promote tumour angiogenesis. These eicosanoids make the tumour microenvironment more favourable for neoplasms and metastasis by encouraging the transcription of angiogenic growth factors, increasing the rate of endothelial cell migration and proliferation, and increasing the rate of vascularization.^38^ It has been reported that higher omega‐6 fatty acids intake was associated with increased risk of BC in women.^39^ Remarkably, it has been reported that carriers of the A allele of *FTO* rs9939609 polymorphism had a higher body fat percentage (BF%).^40^ Timpson et al. in a cohort study reported that people with the rs9939609 minor allele had a higher intake of fat than those with the TT genotype.^41^ Furthermore, we found in previous studies that homozygotes for the rs9939609 risk allele (A) had higher serum leptin compared to the TT genotype.^42^, ^43^ Also, it has been shown that carriers of the AA genotype of rs9939609 had significantly higher calorie, fat and carbohydrate intake than the carriers of the TT genotype after adjusting for age and sex (*p* = 0.019, *p* = 0.010 and *p* = 0.001, respectively).^44^ Moreover, *FTO* gene polymorphism (AA genotype) influence on gut hormones such as PYY3–36 and acyl‐ghrelin levels that lead to increased food intake especially energy‐dense foods and reduced satiety.^45^, ^46^ In rs9939609 AA carriers, suppression of acylated ghrelin led to overeating and obesity.^47^ In addition, it was indicated that *FTO* is involved in lipid metabolism through m6‐A‐mediated epigenetic regulation. It has been shown that *FTO* regulates lipogenesis by altering m6‐A levels of fatty acid synthase (FASN), the key metabolic multi‐subunit enzyme that is responsible for the synthesis of fatty acid.^48^ A growing body of evidence suggests that *FTO* plays critical roles in both overweight/obesity and cancers via m^6^A demethylase of target genes, which affecting the stability and/or splicing of their mRNAs, in turn leading to promoting adipogenesis, tumorigenesis and drug resistance of cancer cells. The strong association between *FTO* SNPs or overweight/obesity with an increased risk of cancers suggests that the obesity‐associated function of *FTO* in metabolism may also contribute to its effects in cancers.^49^ Therefore, the higher intake of omega‐6 in individuals with the risk allele of *FTO* rs9939609 and its positive association with BC risk in those individuals, as shown in the present study, indicates that the association between omega‐6 fatty acids and BC risk can be influenced by *FTO* rs9939609 polymorphism.

This study enhances our understanding of the interaction between genetic susceptibility and dietary intake on cancer risk through assessing dietary intake, cancer risk and *FTO* polymorphism. However, we had some limitations in the present study. First, different types of BC including the status of hormone receptors and also the stage of BC were not considered. Second, this study was limited to only one SNP of the *FTO* gene and other SNPs, and genes may have associations with BC. Finally, the participant sample size was relatively small, and further studies with larger sample sizes are warranted.

## 5 CONCLUSION

The results of this case–control study showed a significant positive association between BC and dietary intake of omega‐6 fatty acids in individuals with risk allele of *FTO* rs9939609 polymorphism. These results suggest that the association between BC and dietary intake can be influenced by *FTO* polymorphism and highlight the interactions of genes and diet on cancer risk. Also, these finding can help strengthen the existing documentation on the need for personalized diets to prevent cancer. Further prospective studies are needed to be carried out on patients with different types of BC to assess the possible effects of the *FTO* genotype on BC risk.

## AUTHOR CONTRIBUTIONS

**Saeid Doaei:** Supervision (equal). **Sepideh Abdollahi:** Software (equal). **Mohammad Esmail Akbari:** Methodology (equal). **Saeed Omidi:** Investigation (equal); validation (equal). **Seyed Mohammad Poorhosseini:** Formal analysis (equal). **Maryam Gholamalizadeh:** Data curation (equal); investigation (equal). **Soudeh Ghafouri‐Fard:** Methodology (equal). **Ghasem Azizi Tabesh:** Formal analysis (equal). **Alireza Moslem:** Visualization (equal). **Naeemeh Hasanpour Ardekanizadeh:** Software (equal). **Seyedeh Elaheh Bagheri:** Validation (equal). **Azita Hekmatdoost:** Validation (equal). **Mahdi Alam Rajabi:** Visualization (equal). **Seyed Alireza Mosavi Jarrahi:** Supervision (equal). **Mark O. Goodarzi:** Validation (equal); writing – review and editing (equal). **Golsa Khalatbari Mohseni:** Data curation (equal); writing – review and editing (equal).

## FUNDING INFORMATION

This study was financially supported by Shahid Beheshti University of Medical Sciences, Tehran, Iran (Code: 74859).

## CONFLICT OF INTEREST

The authors declare that they have no competing interests.

## Supporting information


Supplementary file 1.

## Data Availability

Datasets used and/or analysed during the current study are available from the corresponding author on reasonable requests.

## References

[1] Soerjomataram I , Allemani C , Voogd A , Siesling S . The Global Burden of Breast Cancer in Women. Breast cancer: Global quality care. Oxford University Press; 2019:1.

[2] Iacoviello L , Bonaccio M , de Gaetano G , Donati MB . Epidemiology of breast cancer, a paradigm of the “common soil” hypothesis. Seminars in Cancer Biology. Elsevier; 2021. 10.1016/j.semcancer.2020.02.010 32087245

[3] Kazeminia M , Salari N , Hosseinian‐Far A , Akbari H , Bazrafshan M‐R , Mohammadi M . The prevalence of breast cancer in Iranian women: a systematic review and meta‐analysis. Indian J Gynecol Oncol. 2022;20(1):1‐9.

[4] Gangadharan P , Shaji A , Vijaykumar D , Kunheri B . Breast cancer trends: global and Indian scenario. Management of early stage breast cancer. Springer; 2021:1‐13.

[5] Gong Z , Ambrosone CB , McCann SE , et al. Associations of dietary folate, vitamins B6 and B12 and methionine intake with risk of breast cancer among African American and European American women. Int J Cancer. 2014;134(6):1422‐1435. 23996837 10.1002/ijc.28466 PMC4161208

[6] Liposits G , Orrevall Y , Kaasa S , Österlund P , Cederholm T . Nutrition in cancer care: a brief, practical guide with a focus on clinical practice. JCO Oncol Pract. 2021;17(7):e992‐e998.

[7] Gómez‐Donoso C , Martínez‐González MÁ , Perez‐Cornago A , Sayón‐Orea C , Martínez JA , Bes‐Rastrollo M . Association between the nutrient profile system underpinning the nutri‐score front‐of‐pack nutrition label and mortality in the SUN project: a prospective cohort study. Clin Nutr. 2021;40(3):1085‐1094. 32768318 10.1016/j.clnu.2020.07.008

[8] De Cicco P , Catani MV , Gasperi V , Sibilano M , Quaglietta M , Savini I . Nutrition and breast cancer: a literature review on prevention, treatment and recurrence. Nutrients. 2019;11(7):1514. 31277273 10.3390/nu11071514 PMC6682953

[9] Skouroliakou M , Grosomanidis D , Massara P , et al. Serum antioxidant capacity, biochemical profile and body composition of breast cancer survivors in a randomized Mediterranean dietary intervention study. Eur J Nutr. 2018;57(6):2133‐2145. 28634625 10.1007/s00394-017-1489-9

[10] Jung SY , Scott PA , Papp JC , et al. Genome‐wide association analysis of proinflammatory cytokines and gene–lifestyle interaction for invasive breast cancer risk: the WHI dbGaP study. Cancer Prev Res. 2021;14(1):41‐54. 10.1158/1940-6207.CAPR-20-0256 PMC7956151 32928877

[11] Faramarzi A , Jahromi MG , Ashourzadeh S , Jalilian N . Metastatic and pathophysiological characteristics of breast cancer with emphasis on hereditary factors. Cent Asian J Med Pharm Sci Innov. 2021;1(3):104‐113.

[12] Lee A , Moon B‐I , Kim TH . BRCA1/BRCA2 pathogenic variant breast cancer: treatment and prevention strategies. Ann Lab Med. 2020;40(2):114‐121. 31650727 10.3343/alm.2020.40.2.114 PMC6822003

[13] Yoshida R . Hereditary breast and ovarian cancer (HBOC): review of its molecular characteristics, screening, treatment, and prognosis. Breast Cancer. 2021;28(6):1167‐1180. 32862296 10.1007/s12282-020-01148-2 PMC8514387

[14] Lee HY , Cha J , Kim SK , et al. c‐MYC drives breast cancer metastasis to the brain, but promotes synthetic lethality with TRAIL. Mol Cancer Res. 2019;17(2):544‐554. 30266755 10.1158/1541-7786.MCR-18-0630

[15] Liu Y‐L , Chou C‐K , Kim M , et al. Assessing metastatic potential of breast cancer cells based on EGFR dynamics. Sci Rep. 2019;9(1):1‐13. 30833579 10.1038/s41598-018-37625-0 PMC6399327

[16] Gholamalizadeh M , Akbari ME , Doaei S , et al. The Association of fat‐Mass‐and Obesity‐Associated Gene Polymorphism (rs9939609) with colorectal cancer: a case‐control study. Front Oncol. 2021;11:3823. 10.3389/fonc.2021.732515 PMC8506030 34650918

[17] Melhorn SJ , Askren MK , Chung WK , et al. FTO genotype impacts food intake and corticolimbic activation. Am J Clin Nutr. 2018;107(2):145‐154. 29529147 10.1093/ajcn/nqx029 PMC6454473

[18] Stratigopoulos G , Padilla SL , LeDuc CA , et al. Regulation of Fto/Ftm gene expression in mice and humans. Am J Physiol Regul Integr Comp Physiol. 2008;294(4):R1185‐R1196. 18256137 10.1152/ajpregu.00839.2007 PMC2808712

[19] Hakanen M , Raitakari OT , Lehtimäki T , et al. FTO genotype is associated with body mass index after the age of seven years but not with energy intake or leisure‐time physical activity. J Clin Endocrinol Metabol. 2009;94(4):1281‐1287. 10.1210/jc.2008-1199 19158205

[20] Antonio J , Knafo S , Kenyon M , et al. Assessment of the FTO gene polymorphisms (rs1421085, rs17817449 and rs9939609) in exercise‐trained men and women: the effects of a 4‐week hypocaloric diet. J Int Soc Sports Nutr. 2019;16(1):1‐9. 31477138 10.1186/s12970-019-0307-6 PMC6719365

[21] Ludwig DS . The glycemic index: physiological mechanisms relating to obesity, diabetes, and cardiovascular disease. JAMA. 2002;287(18):2414‐2423. 11988062 10.1001/jama.287.18.2414

[22] Hernández‐Caballero ME , Sierra‐Ramírez JA . Single nucleotide polymorphisms of the FTO gene and cancer risk: an overview. Mol Biol Rep. 2015;42(3):699‐704. 25387436 10.1007/s11033-014-3817-y

[23] Delahanty RJ , Beeghly‐Fadiel A , Xiang Y‐B , et al. Association of obesity‐related genetic variants with endometrial cancer risk: a report from the Shanghai endometrial cancer genetics study. Am J Epidemiol. 2011;174(10):1115‐1126. 21976109 10.1093/aje/kwr233 PMC3246689

[24] Kaklamani V , Yi N , Sadim M , et al. The role of the fat mass and obesity associated gene (FTO) in breast cancer risk. BMC Med Genet. 2011;12(1):1‐10. 21489227 10.1186/1471-2350-12-52 PMC3089782

[25] Lin Y , Ueda J , Yagyu K , et al. Association between variations in the fat mass and obesity‐associated gene and pancreatic cancer risk: a case–control study in Japan. BMC Cancer. 2013;13(1):1‐6. 23835106 10.1186/1471-2407-13-337 PMC3716552

[26] Huang X , Zhao J , Yang M , Li M , Zheng J . Association between FTO gene polymorphism (rs9939609 T/a) and cancer risk: a meta‐analysis. Eur J Cancer Care. 2017;26(5):e12464. 10.1111/ecc.12464 26931363

[27] Ağagündüz D , Gezmen‐Karadağ M . Association of FTO common variant (rs9939609) with body fat in Turkish individuals. Lipids Health Dis. 2019;18(1):1‐12. 31810473 10.1186/s12944-019-1160-y PMC6896279

[28] Lan N , Lu Y , Shuangshuang P , et al. FTO‐a common genetic basis for obesity and cancer. Front Genet. 2020;11:1149. 10.3389/fgene.2020.559138 PMC7701174 33304380

[29] Gholamalizadeh M , Jarrahi AM , Akbari ME , et al. Association between FTO gene polymorphisms and breast cancer: the role of estrogen. Expert Rev Endocrinol Metab. 2020;15(2):115‐121. 32089015 10.1080/17446651.2020.1730176

[30] Jia T , Liu Y , Fan Y , Wang L , Jiang E . Association of healthy diet and physical activity with breast cancer: lifestyle interventions and oncology education. Front Public Health. 2022;10. 10.3389/fpubh.2022.797794 PMC8984028 35400043

[31] Doaei S , Bourbour F , Rastgoo S , et al. Interactions of anthropometric indices, rs9939609 FTO gene polymorphism and breast cancer: a case‐control study. J Cell Mol Med. 2021;25(7):3252‐3257. 33634577 10.1111/jcmm.16394 PMC8034447

[32] De Lorgeril M , Salen P . New insights into the health effects of dietary saturated and omega‐6 and omega‐3 polyunsaturated fatty acids. BMC Med. 2012;10(1):1‐5. 22613931 10.1186/1741-7015-10-50 PMC3394202

[33] Dydjow‐Bendek D , Zagoźdźon P . Total dietary fats, fatty acids, and Omega‐3/Omega‐6 ratio as risk factors of breast cancer in the polish population—a case‐control study. In Vivo. 2020;34(1):423‐431. 31882509 10.21873/invivo.11791 PMC6984116

[34] Nindrea RD , Aryandono T , Lazuardi L , Dwiprahasto I . Association of dietary intake ratio of n‐3/n‐6 polyunsaturated fatty acids with breast cancer risk in Western and Asian countries: a meta‐analysis. Asian Pac J Cancer Prev. 2019;20(5):1321‐1327. 31127884 10.31557/APJCP.2019.20.5.1321 PMC6857870

[35] Peppone LJ , Inglis JE , Mustian KM , et al. Multicenter randomized controlled trial of omega‐3 fatty acids versus omega‐6 fatty acids for the control of cancer‐related fatigue among breast cancer survivors. JNCI Cancer Spectr. 2019;3(2):pkz005. 31119206 10.1093/jncics/pkz005 PMC6512349

[36] Welsch CW . Relationship between dietary fat and experimental mammary tumorigenesis: a review and critique. Cancer Res. 1992;52(7 Supplement):2040s‐2048s. 1544139

[37] Huerta‐Yépez S , Tirado‐Rodriguez AB , Hankinson O . Role of diets rich in omega‐3 and omega‐6 in the development of cancer. Bol Med Hosp Infant. 2016;73(6):446‐456. 10.1016/j.bmhimx.2016.11.001 29421289

[38] Kang JX , Liu A . The role of the tissue omega‐6/omega‐3 fatty acid ratio in regulating tumor angiogenesis. Cancer Metastasis Rev. 2013;32(1):201‐210. 23090260 10.1007/s10555-012-9401-9

[39] Gholamalizadeh M , Shahdoosti H , Bahadori E , et al. Association of different types of dietary fatty acids with breast cancer, a case‐control study. Nutr Food Sci. 2021;52:561‐568.

[40] Gholamalizadeh M , Mirzaei Dahka S , Vahid F , et al. Does the rs9939609 FTO gene polymorphism affect fat percentage? A meta‐analysis. Arch Physiol Biochem. 2020;1‐5. 10.1080/13813455.2020.1773861 32574121

[41] Timpson NJ , Emmett PM , Frayling TM , et al. The fat mass–and obesity‐associated locus and dietary intake in children. Am J Clin Nutr. 2008;88(4):971‐978. 18842783 10.1093/ajcn/88.4.971 PMC4773885

[42] Mehrdad M , Doaei S , Gholamalizadeh M , Fardaei M , Fararouei M , Eftekhari MH . Association of FTO rs9939609 polymorphism with serum leptin, insulin, adiponectin, and lipid profile in overweight adults. Adipocyte. 2020;9(1):51‐56. 31996075 10.1080/21623945.2020.1722550 PMC6999843

[43] Jalili V , Mokhtari Z , Rastgoo S , et al. The association between FTO rs9939609 polymorphism and serum lipid profile in adult women. Diabetol Metab Syndr. 2021;13(1):1‐6. 34801066 10.1186/s13098-021-00754-0 PMC8606052

[44] Mehrdad M , Doaei S , Gholamalizadeh M , Eftekhari MH . The association between FTO genotype with macronutrients and calorie intake in overweight adults. Lipids Health Dis. 2020;19(1):1‐6. 32843047 10.1186/s12944-020-01372-x PMC7449073

[45] Wardle J , Carnell S , Haworth CM , Farooqi IS , O'Rahilly S , Plomin R . Obesity associated genetic variation in FTO is associated with diminished satiety. J Clin Endocrinol Metabol. 2008;93(9):3640‐3643. 10.1210/jc.2008-0472 18583465

[46] Velders FP , De Wit JE , Jansen PW , et al. FTO at rs9939609, food responsiveness, emotional control and symptoms of ADHD in preschool children. PloS One. 2012;7(11):e49131. 23155456 10.1371/journal.pone.0049131 PMC3498333

[47] Karra E , O'Daly OG , Choudhury AI , et al. A link between FTO, ghrelin, and impaired brain food‐cue responsivity. J Clin Invest. 2013;123(8):3539‐3551. 23867619 10.1172/JCI44403 PMC3726147

[48] Sun D , Zhao T , Zhang Q , Wu M , Zhang Z . Fat mass and obesity‐associated protein regulates lipogenesis via m6A modification in fatty acid synthase mRNA. Cell Biol Int. 2021;45(2):334‐344. 33079435 10.1002/cbin.11490

[49] Deng X , Su R , Stanford S , Chen J . Critical enzymatic functions of FTO in obesity and cancer. Front Endocrinol. 2018;9:396. 10.3389/fendo.2018.00396 PMC6077364 30105001

