# Supplementary material for: The effects of FTO gene rs9939609 polymorphism on the association between breast cancer and dietary intake
Source: J Cell Mol Med. 2022 Nov 20;26(23):5794–806. doi: 10.1111/jcmm.17595 (PMC9716323; doi:10.1111/jcmm.17595)
Supplement: Supplementary file 1 [file JCMM-26-5794-s001.docx]

Supplementary file 1. The features of FTO gene primers used in this study.

| **Primer** | **Primer sequence** | **Product size** | **Annealing temp.** |
| --- | --- | --- | --- |
| F outer | AGTTCCAGTCATTTTTGACAGC | 429 | 57.43 |
| R outer | AGCCTCTCTACCATCTTATGTC |  | 56.51 |
| F inner | CCTTGCGACTGCTGTGAATATA | 278 | 58.54 |
| R inner | GAGACTATCCAAGTGCATCTCA |  | 57.34 |
